# Get screened: a pragmatic randomized controlled trial to increase mammography and colorectal cancer screening in a large, safety net practice

**DOI:** 10.1186/1472-6963-10-280

**Published:** 2010-09-23

**Authors:** Kevin Fiscella, Amanat Yosha, Samantha K Hendren, Sharon Humiston, Paul Winters, Pat Ford, Starlene Loader, Raymond Specht, Shirley Pope, Amna Adris, Steven Marcus

**Affiliations:** 1 Department of Family Medicine and Community & Preventive Medicine, University of Rochester; 1381 South Ave, Rochester, NY 14620, USA; 2 Departments of Family Medicine, University of Rochester; 1381 South Ave, Rochester, NY 14620, USA; 3 Department of Surgery, University of Michigan, 2124 Taubman Center, 1500 East Medical Center Drive SPC-5343, Ann Arbor, MI 48109, USA; 4 Emergency Medicine Services Division, Children's Mercy Hospitals and Clinics; 2401 Gillham Road, Kansas City, MO 64108, USA; 5 Departments of Family Medicine, Highland Hospital; 1381 South Ave, Rochester, NY 14620, USA

## Abstract

**Background:**

Most randomized controlled trials of interventions designed to promote cancer screening, particularly those targeting poor and minority patients, enroll selected patients. Relatively little is known about the benefits of these interventions among unselected patients.

**Methods/Design:**

"Get Screened" is an American Cancer Society-sponsored randomized controlled trial designed to promote mammography and colorectal cancer screening in a primary care practice serving low-income patients. Eligible patients who are past due for mammography or colorectal cancer screening are entered into a tracking registry and randomly assigned to early or delayed intervention. This 6-month intervention is multimodal, involving patient prompts, clinician prompts, and outreach. At the time of the patient visit, eligible patients receive a low-literacy patient education tool. At the same time, clinicians receive a prompt to remind them to order the test and, when appropriate, a tool designed to simplify colorectal cancer screening decision-making. Patient outreach consists of personalized letters, automated telephone reminders, assistance with scheduling, and linkage of uninsured patients to the local National Breast and Cervical Cancer Early Detection program. Interventions are repeated for patients who fail to respond to early interventions. We will compare rates of screening between randomized groups, as well as planned secondary analyses of minority patients and uninsured patients. Data from the pilot phase show that this multimodal intervention triples rates of cancer screening (adjusted odds ratio 3.63; 95% CI 2.35 - 5.61).

**Discussion:**

This study protocol is designed to assess a multimodal approach to promotion of breast and colorectal cancer screening among underserved patients. We hypothesize that a multimodal approach will significantly improve cancer screening rates.

The trial was registered at Clinical Trials.gov NCT00818857

## Background

Poor, underserved-minority, and uninsured patients have lower rates of cancer screening than other Americans. Lower rates of screening contribute to disparities in cancer mortality. For example, African Americans have higher incidence of colorectal cancer (CRC), yet are screened at lower rates. This represents an example of the inverse care law (similar to the treatment-risk paradox[1]) whereby those with the greatest health care need (in this case higher need for screening) are least likely to receive care[2].

This unfortunate paradox in cancer screening likely results from a combination of fewer resources available to patients and fewer resources available to safety net practices serving poor, minority and/or uninsured patients. Underserved patients often face insurance[3,4] and other financial barriers,[5] in addition to barriers related to knowledge,[6,7] mistrust,[8] limited English proficiency (LEP),[9] self efficacy, [10-12] and health care literacy[13]. Underserved patients are more likely to be cared for in under-resourced practices[14-16].

There are two key challenges to development of interventions that improve cancer screening among underserved patients. The first challenge is conceptual and the second is methodological - sample selection bias. The conceptual challenge results from the finding that low-intensity cancer screening promotion interventions may not necessarily help close gaps in cancer screening, particularly if harder-to-reach groups respond at lower rates to these interventions. For example, a single reminder mailed out by health plans to patients who are soon due for cancer screening yields higher screening rates for patients with greater resources resulting in increased screening disparities[17].

One way to address the unintended consequence of increasing disparities is to invert the inverse care law and allocate greater resources to patients who face greater screening barriers. This approach has been successfully used to eliminate community-wide disparities in other preventive services, particularly immunizations[18]. In this case, Szilagyi et al implemented reminders, recall and outreach within safety net practices. They compiled practice-based registries, *among all eligible patients *(not a selected subsample) and implemented a combination of in-reach (clinician prompts at the time of patient visits) and outreach to patients past due using increasingly intensive interventions (letter, phone calls and even home visits) to families that failed to respond to less intensive interventions. We derived principles from their project and applied these principles to cancer screening.

### Principles for addressing disparities in cancer screening

We adapted the following four principles to promotion of mammography and colorectal cancer screening:

#### 1) Patients needing clinical services are identified using data from primary care practices

Although this approach does not reach all persons, i.e. those not registered at a primary care practice, it better targets resources to unscreened persons than broad-based community approaches. Also, most randomized controlled trials formally enroll patients, leading to sample selection bias because harder-to-reach patients are more difficult to reach for enrollment. Results from these trials - particularly those involving outreach - may not readily apply to harder-to-reach groups. By identifying patients from primary care practices and embedding the intervention in the care of randomly selected patient care, we skirt this potential bias.

#### 2) Resources are focused on practices that care for large numbers of underserved patients

Underserved patients often cluster within relatively few practices; focusing on these practices directs resources to where they are needed the most.

#### 3) Personal physician recommendations are leveraged to promote preventive services

Physicians almost universally endorse cancer screening[19]. However, high rates of physician recommendations for screening are not supported by either chart documentation [20] or patient report[21]. Clinicians' apparent failure to recommend cancer screening despite their best intentions is likely attributable to competing demands for their time and attention during busy offices visits,[22,23] such as management of multiple acute problems, chronic illnesses, psychosocial problems, completion of documentation, referrals, and phone calls. Competing demands reduce rates of the delivery of preventive services,[24,25] including cancer screening[26]. Prompting clinicians may help,[27,28] but is only relevant to patients who come in for care and clinicians may inadvertently disregard prompts[29]. Patient prompts combined with outreach to patients may complement clinician prompts at point-of-care.

#### 4) Outreach directs increasingly intensive interventions to those who have failed to respond to earlier interventions

This focuses more intensive resources on those who are harder-to-reach.

This project aims to address both conceptual and sample selection limitations. It adopts a multimodal approach to screening promotion and evaluates it through a randomized controlled trial with minimal selection bias in enrollment.

## Methods/Design

### Study design

We use a pragmatic, randomized controlled trial to evaluate this multimodal intervention with the patient as the unit of randomization. This allows for direct comparison of cancer screening rates (intervention vs. control groups) among patients who at the outset are past due for mammography or colorectal cancer screening. We chose to randomize at the patient level rather than physician or physician group level because it is difficult to ensure comparability when randomizing relatively small numbers of physicians or physician groups. It should be noted that patient-level randomization allows for physician learning effects, which bias results towards the null so results from this trial may be conservative.

**Figure 1:**
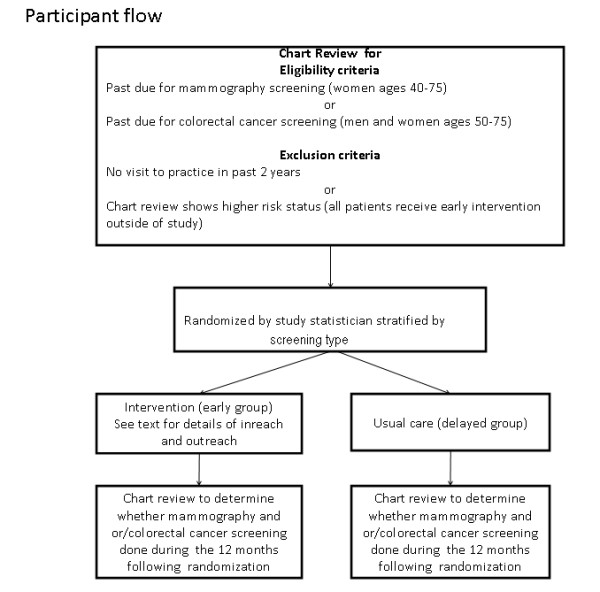
**Patient flow**.

The study design is shown in Figure 1. Individual patients are randomized and receive the 6 month intervention. Twelve months following randomization the delayed group also receives intervention. The primary analysis will be based on comparison of screening rates of patients in the early group who receive the intervention with those in the delayed group who receive usual care during the same time period. To account for lags between referral and completion of screening, rates of screening will be compared over 12 months.

### Selection of practice site

We recruited one large safety net practice in Rochester, NY to participate in this trial. Given our aim of evaluating the impact of this multimodal intervention on disparities in cancer screening, we targeted a practice that serve large numbers of low-income and minority patients.

### Participant consent, eligibility and randomization

The study was approved by the Institutional Review Board of the University of Rochester. Participant informed consent was waived by the Institutional Review Board because the study poses no greater risk to participants than that associated with routine care, participants are free to ignore in-reach or outreach and may request that they receive no further reminders at any time.

Participant eligibility criteria for enrollment in the study include: (1) being a registered patient at the practice (we used a single download from the health system to create a practice registry); (2) having at least one visit to the practice in the past two years (to ensure participants were actively receiving care at the practice); (3) being ages 40-75 years for mammography screening, and 50-75 years for CRC screening; (4) being past due for annual mammography or CRC screening [(recommended intervals are 10 years for those screened through colonoscopy, 5 years for those screened with sigmoidoscopy and/or barium enema, and annually for those screened through fecal occult blood tests (FOBT)]. Women ages 50-75 could be enrolled based on either or both mammography and CRC criteria. Patients at higher risk for cancer due to prior cancer, premalignant conditions, inadequately evaluated breast mass, or positive FOBT were not randomized. Higher risk patients were excluded from randomization due to ethical concerns; clinicians were notified if chart review revealed they were overdue for screening.

Potentially eligible participants were identified using practice billing data based on age, sex and last visit to the practice. These data, in addition to race, ethnicity, presence and type of insurance, name of the primary care physician, and contact information were imported into a secure, customized patient tracking registry that was created in Microsoft Access^®^. Last dates of screening were determined through manual review of the electronic medical record (EMR) by research assistants using structured abstract forms to determine if, when and how participants were last screened.

After a research assistant determined eligibility, the off-site, statistician - blinded to all patient information not needed for stratification - assigned patients to early (intervention) or delayed (control) intervention using computer-generated random numbers. Randomization was stratified by screening type (mammography or CRC) to ensure that comparable groups of patients are randomized to each arm. Unique ID numbers were assigned to patients that identify their intervention group. The statistician maintained the key; all other study personnel were blinded to the intervention group assignment. The encoded sequences were affixed to the Case Report File (CRF) as well as the patient chart. Patients in both the intervention and control groups are followed for one year.

### Description of the intervention

The intervention, referred to as "Get Screened," builds on the finding that multimodal initiatives are more powerful for promoting use of preventive services[30]. We use paper prompts that are delivered to the clinician and patient immediately prior to the visit by office medical assistants. No more than three prompts are delivered for the same patient. One research assistant uses the EMR to indentify unscreened patients in the intervention group who have impending visits in during the week. She organizes patients by day of week into an accordion folder and delivers this to each suite within the large practice.

#### Clinician Prompt

We designed clinician-friendly, paper prompts that indicate that the patient is past due for mammography and/or CRC screening. We piloted the prompt sheet and obtained feedback from clinicians in the practice. CRC screening involves a number of different potential screening modalities[31]. Based on our discussions with community practices and local imaging centers, we have found that FOBT and colonoscopy were primarily used; flexible sigmoidoscopy, double contrast barium enema, and virtual colonoscopy were seldom used to screen for CRC locally. The back of each prompt sheet includes a brief summary of the relative advantages and limitations for each screening modality intended to facilitate clinician-patient discussion. The outreach worker also delivers fecal immunochemical testing kits to the practice and instructs medical assistants in how to counsel patients in their use and how to complete the test requisition. Fecal immunochemical tests and referral forms for colonoscopy are made available at the time of the patient visit.

#### Patient Prompts

We designed a simple patient prompt based on a low-literacy tool used to prompt patients to request pneumococcal vaccination[32]. We piloted the tool and received suggestions from our community-advisory board.

#### Patient Outreach

Outreach to unscreened patients consists of two personalized letters and up to four automated telephone reminder (ATR) calls. The letter is signed by the patient's primary care clinician and indicates that the patient is overdue for screening and why screening is important. It includes relevant phone numbers including how uninsured patients can obtain free screening. The automated telephone reminders are scripted, pre-recorded messages that include the patient's first name. The message identifies the caller and the practice; it then informs the patient s/he is past due and the phone number to call to schedule a screening (mammography) or an appointment (to discuss CRC). A completed call is defined as an answered call (either in person or a machine).

The first letter is sent within the first week of enrollment. This is followed by two completed ATRs at week 2 and 6 [33,34]. For patients who remain unscreened, a second letter is mailed out at week 12 followed by a third ATR at week 14. For patients past due for CRC screening, the letter includes a testing kit for fecal immunochemical testing for home use. A final ATR is made at week 26.

Both the letters and ATRs provide the phone number of the outreach worker if help is needed. Using a three way call option, the outreach worker can link patients with mammography schedulers or with the National Breast and Cervical Cancer Early Detection Program (NBCCEP), which provides free screening for the uninsured[35].

### Creation of patient tracking registry

We were unable to identify a commercially available tracking registry that met our study requirements. Hence, we designed our own registry using Microsoft Access^®^. The key functionalities of the registry include:

1) Electronic importation of data from billing data (patient name, age, gender, race, ethnicity, insurance type, primary care clinician, contact information, date of last patient visit).

2) Location on a secure university web server and restricted access to different fields based on unique passwords. For example, the outreach worker only has access to data for patients assigned to the intervention. Similarly, research assistants are blinded to group assignment and related data.

3) Tracking includes the date of all interventions (prompts, letters and phone calls) and date and type of cancer screening.

4) Report writing - ability to generate reports on patients based on types and dates of interventions received or other key fields.

5) Patient screening test results are used to update the registry facilitating removal of screened patients from further intervention.

### Description of usual care

All patients randomized to the delayed intervention receive "usual care" during the twelve months following randomization. In this practice, the clinician (family physician, nurse practitioner, or physician assistant) is responsible for discussing cancer screening with the patient and for initiating any referral or for handing out FOBT cards. The EMR has a section called health maintenance profile that allows for input of the last screening date to be entered and generation of alerts for future screening. However, discussion with clinicians suggests that this feature is not clinician-friendly and is seldom used due to time constraints. Instead, each clinician has his/her own method for tracking cancer screening. In our pilot site, many clinicians reported not having organized systems for contacting patients overdue for screening outside of scheduled visits.

### Baseline and follow-up measures

Table 1 lists the baseline measures abstracted by a research assistant who is blinded to group assignment. The primary outcome measure is medical record documentation of completed mammography or any type of CRC screening during the 12 months following randomization. Covariates including age, gender, race, ethnicity, insurance, number of visits in year prior to randomization and comorbidity (based on the number of problems listed on patient problem lists) will be used to compare key characteristics of early and delayed groups and test for interactions.

**Table 1:** Baseline measures

*Category*	*Baseline Measure*
Demographics	Age (years), sex (male/female, race (White, African American/Black, Asian other), Hispanic ethnicity (yes/no), and insurance (yes/no) and if yes

Insurance	Any (Yes/No) Type (Private, Medicare, Medicaid), HMO (Yes/No)

Visit Utilization	Number of visits in year preceding randomization

Comorbidity	Number of conditions listed on patient problem list

Cancer risk	Breast cancer risk factors (prior breast cancer, BCRA, family history) CRC risk factors (Prior polyp, inflammatory bowel disease, genetic condition)

Cancer screening history	Date of last mammogram and results (Normal/Abnormal) Date, type, and results (Normal/Abnormal) of CRC screening: Colonoscopy FOBT Sigmoidoscopy Double contrast barium enema

### Role of the community health worker

The CHW functions as both an outreach worker and practice facilitator. S/he uses the patient registry to identify patients who are unscreened and implement outreach either mailing out letters or responding to phone calls to patients, and mailing out FOBT kits to unscreened patients. S/he functions as a practice facilitator by working with each practice to implement a tailored system for reminders to patients and clinicians, and also to facilitate referrals of patients who request them for mammography or colonoscopy.

### Preliminary data

In 2009, we piloted the intervention using the eligibility criteria described above. In the mammography arm, 270 women met eligibility criteria for enrollment and 323 men and women met eligibility criteria for enrollment in the CRC arm. The sample was middle aged (22% 40-49 years, 48% 50-59 years, and 29% 60 years and older) and 70% female. Distribution of race/ethnicity was 61% White, 28% Black, 5% Hispanic, and 5% Asian. Health insurance distribution was 42% private (including supplemental private insurance for Medicare), 25% Medicaid, 23% Medicare only, and 9% uninsured.

For the pilot study (but not for the main study) patients were assigned to early v. delayed intervention based on their medical record number. The characteristics of the two groups were similar. The pilot results are notable in two respects. First, patients who were past due had very low rates of screening in the subsequent 12 months. Second, rates of screening were three times higher in the intervention compared to the control group. Because the patients were not randomized, but inadvertently assigned based on medical record number, we conducted a logistic regression model to predict screening that controlled for participant characteristics. These adjusted results from the pilot were similar to our crude results from the pilot study (Odds Ratio 3.63; 95% CI 2.35- 5.61) for having any screening.

### Sample Size determination

For the main study, based on the size of the recruited practice and screening rates from the pilot study, we estimate that 190 patients will be past due for mammography and 239 will be past due for CRC screening. Based on our pilot data, we anticipate rates of screening of 18% and 10% respectively in the usual care (delayed group). Based on our pilot data, we conservatively estimate that this multimodal intervention will double screening rates for both mammography and CRC screening among those in the early intervention group. Based on these assumptions, we will have a minimum of 80% power to detect a difference of 18% in the mammography group and 13% in the colorectal screening group using 95% confidence intervals.

### Patient accrual and study flow

Patient flow for the main study is shown in figure 1. Of the 525 women 40-75 years and men 50-75 years of age that had a visit during the past two years, 148 had undergone timely cancer screening. Of these and 10 were deemed higher risk and not eligible for randomization. A total of 367 patients will be randomized to the two groups.

### Planned analytic approach

Intervention vs. control groups will be compared based on intention-to-treat. That is, once randomized patients will be included in the analysis regardless of whether they leave the practice or not. We will use Generalized Estimating Equation models to control for covariates and clustering within practices. We also will assess whether expected baseline differences in screening by race, ethnicity and insurance are attenuated in the intervention group. We will use a difference in difference approach to assess whether the differences between Whites and Blacks, Hispanics and non-Hispanics, and insured and uninsured patients are significantly smaller in the intervention (early) compared to usual care (delayed group).

## Discussion

"Get Screened" is a pragmatic, randomized controlled trial of a multi-modal intervention designed to improve mammography and CRC screening among unselected, low-income patients. This intervention design builds upon prior success in the field of immunization in which multimodal, increasingly intensive interventions improved immunizations across various patient characteristics (e.g. race, ethnicity, and insurance) in safety net practices and decreased disparities. Preliminary data during the pilot study suggest that this multimodal intervention could triple rates of screening in practices with low baseline rates of screening. We hope to confirm these promising findings using a randomized, controlled study design.

Other notable features of the project include patient-level randomization, targeting of safety-net practices, creation of a practice-based registry and a combination of clinician and patient prompts at point-of-care combined with increasingly intensive outreach to unscreened patients.

Key challenges during early stages of implementation have included limitations of the EMR, especially the lack of an electronic alert system that clinicians actually use. The finding that the EMR was poorly designed for tracking preventive health services and providing reminders, led to modification of our alert intervention plan. It also presented an opportunity to populate this portion of the EMR in order to promote future cancer screening tracking within the practice. It is likely that many EMR systems are similarly limited in the degree of practical help they provide clinicians in improving rates of cancer screening.

Another challenge involves developing an efficient system for identifying patients who are screened during the intervention period in order to focus resources on those remaining unscreened. The complex range of CRC screening choices was cited by participating clinicians as a barrier to discussion of screening. This and other feedback from the practice was used during early pilot work to modify and tailor the intervention. One modification was the use of a simplified CRC point-of-care decision-making tool that focused on the choice between colonoscopy and FOBT testing. Findings from this study should inform implementation of cancer screening within safety net primary care practices.

## Competing interests

The authors declare that they have no competing interests.

## Authors' contributions

KF lead the design and directed the project, and manuscript writing. AY, SKH, SH, contributed to the protocol design and writing, PW contributed to the statistical analysis, PF is project coordinator and contributed to data collection and analysis, SL contributed to the protocol design, RS, SP, AA, and SM contributed to protocol design, project implementation and data collection. All the authors read and approved the final manuscript.

## Pre-publication history

The pre-publication history for this paper can be accessed here:

http://www.biomedcentral.com/1472-6963/10/280/prepub
